# Aberrant Sporogonic Development of Dmc1 (a Meiotic Recombinase) Deficient *Plasmodium berghei* Parasites

**DOI:** 10.1371/journal.pone.0052480

**Published:** 2012-12-21

**Authors:** Godfree Mlambo, Isabelle Coppens, Nirbhay Kumar

**Affiliations:** 1 Department of Molecular Microbiology and Immunology, Bloomberg School of Public Health, Johns Hopkins University, Baltimore, Maryland, United States of America; 2 Department of Tropical Medicine, School of Public Health and Tropical Medicine, Tulane University, New Orleans, Louisiana, United States of America; Museum National d’Histoire Naturelle, France

## Abstract

**Background:**

In *Plasmodium*, meiosis occurs in diploid zygotes as they develop into haploid motile ookinetes inside the mosquito. Further sporogonic development involves transformation of ookinetes into oocysts and formation of infective sporozoites.

**Methodology/Principal Findings:**

Reverse genetics was employed to examine the role of the meiotic specific recombinase Dmc1, a bacterial RecA homolog during sporogony in *Plasmodium berghei*. PbDmc1 knockout (KO) parasites showed normal asexual growth kinetics compared to WT parasites; however oocyst formation in mosquitoes was reduced by 50 to 80%. Moreover, the majority of oocysts were retarded in their growth and were smaller in size compared to WT parasites. Only a few Dmc1 KO parasites completed maturation resulting in formation of fewer sporozoites which were incapable of infecting naive mice or hepatocytes *in vitro*. PbDmc1 KO parasites were shown to be approximately 18 times more sensitive to Bizelesin, a DNA alkylating drug compared to WT parasites as reflected by impairment of oocyst formation and sporogonic development in the mosquito vector.

**Conclusions/Significance:**

Our findings suggest that PbDmc1 plays a critical role in malaria transmission biology.

## Introduction

*Plasmodium* parasites, causative agents of malaria undergo mitotic and meiotic division at several stages of their life-cycle. Though mitosis takes place during hepatic, erythrocytic and sporogonic (oocyst) development, meiosis only occurs when parasites develop sexually in the mosquito during differentiation and development of diploid zygotes to haploid ookinetes. DNA recombination occurs during mitosis/meiosis and is important for generating genetic diversity in the parasite (Figure S1). However, the molecular mechanisms of DNA recombination in *Plasmodium* are not well characterized [1]. Formation of ookinetes after meiotic division of zygotes represents a bottle neck in the development of *P. falciparum* parasites, i.e out of thousands (range, 1–100,000) of gametocytes (precursors of gametes) taken by mosquitoes during a blood meal only a few ookinetes (∼5) are formed per individual mosquito [2]. Any intervention targeting meiotic recombination in these developmental stages can potentially reduce transmission of malaria to the vertebrate host.

Meiotic division and recombination are critical in sexually reproducing organisms to maintain the same number of chromosomes per daughter cell in each successive generation and to generate genetic diversity [3]. In diploid organisms, meiotic division is essential to maintain the genome size by halving chromosomes in gametes. However, in haploid organisms such as *Plasmodium* parasites, meiosis is required to maintain a haploid genome soon after the formation of a diploid zygote. In *Plasmodium*, meiosis completes before the formation of mature ookinetes and an ookinete nucleus contains four distinct haploid genomes which could be of parental origin or recombinants of the parental types [2], [4]. Examination of oocysts (a stage of the parasite formed from differentiated ookinetes) by dissecting and genotyping individual oocysts has provided evidence for the existence of up to 4 different genotypes within a single oocyst [5].

Dmc1 (disrupted meiotic cDNA) and Rad51 are bacterial RecA homologs and play a role in mitosis and meiosis [6]. Both Dmc1 and Rad51 are structurally similar and are widely conserved among several species. Dmc1 and Rad51 colocalize during meiosis to form nuclear complexes before meiotic chromosome synapses that is essential to stabilize ssDNA and promote recombination [7]. During bacterial recombination, RecA binds to ssDNA to form a nucleoprotein filament. The RecA-ssDNA complex allows the search for homologous sequences and catalyzes exchange of the DNA strand [8]. Rad51 and Dmc1 are important in meiotic recombination and Dmc1 was first described in *Saccharomyces cerevisiae* when mutants were screened for the ability to form spores [9]. ScDmc1 mutants were deficient in chromosome synapses and arrested during meiotic prophase suggesting that Dmc1 plays a central function during meiotic recombination [9].

During meiosis, DNA is duplicated once followed by two rounds of chromosome segregation. In the first step, homologous chromosomes pair and exchange genetic information and in the second step, sister chromatids separate to create four haploid genomes. As best understood in *S. cerevisiae,* meiotic recombination involves repair of DNA double stranded breaks (DSB) that are induced by the protein Spo11 [10]. Following cleavage of DNA by Spo11, exonucleases process the DNA to produce 3′ single stranded tails that are substrates for DNA binding proteins such as Dmc1 and Rad51. The 3′ single stranded tails can be as long as 1,000 bp in size [3]. Dmc1, Rad51 and other nuclear proteins stabilize the single stranded 3′ tails and catalyze strand exchange with the homologous chromosome. When DBS are repaired there are two possible outcomes, the first one results in exchange of DNA between homologous chromosomes also known as cross-over and the second outcome results in no exchange of genetic information or non-crossover [11]. The process of meiotic recombination is tightly regulated and not all regions of the chromosome are involved in recombination [11]. Any error resulting from improperly repaired DSB may have detrimental effects on the cell.

Though the molecular machinery involved in DNA recombination is poorly understood, in *Plasmodium falciparum* there are several lines of evidence that support gene recombination. The genetic diversity observed in genes encoding antigens such as Msp-1 and AMA-1 is a result of genetic recombination [12], [13], [14], [15]. The repertoire of the antigen variant gene family (*var* genes) is maintained by intragenic recombination [16], [17], [18], [19]. Further support for involvement of recombination machinery in *P. falciparum* comes from the success in obtaining gene knockouts resulting from integration of episomally introduced DNA in the parasite [20], [21].

Rad51 was recently identified in *P. falciparum* where it is highly expressed in a methyl methane sulfonate (MMS, a DNA alkylating agent) inducible manner in schizonts, a stage that is mitotically active [1] and nothing is known so far about the role of Dmc1 in *Plasmodium* meiotic recombination. In this report we applied reverse genetics to examine the role of Dmc1 in *P. berghei*, a rodent malaria parasite. An understanding of the molecular role of PbDmc1 during meiosis may identify novel mechanisms involved in the generation of genetic diversity in the malaria parasite population and provide a rationale for the development of novel chemotherapeutics against malaria transmission.

## Results

### Identification of Dmc1 in *P. berghei*

A blast analysis of the *Plasmodium* spp genomic database (www.plasmodb.org) with *S. cerevisiae* Dmc1 sequence (Gene accession number NC_001137) revealed a high score 844 (49% identity and, 69% similarity) with a gene annotated as a putative meiotic recombination protein Dmc1 in *P. berghei* (gene ID PB000874.00.0) and with Dmc1 from *P. falciparum* with a score of 816 (48% identities and 69% positives, gene ID MAL8P1.76). Dmc1 has homologues in the human (*P. vivax*), non-human primate (*P. knowlesi*) and rodent (*P.yoelii, P. chabaudi*) malaria parasites (Table S1). In *P. berghei*, Dmc1 gene (6 introns) consists of a 1035 bp open reading frame that codes for 345 amino acids. The Dmc1 protein has two domains, the N-terminal DNA binding domain and the ATP binding domain.

### Targeted Disruption of PbDmc1 Gene

**Figure 1:**
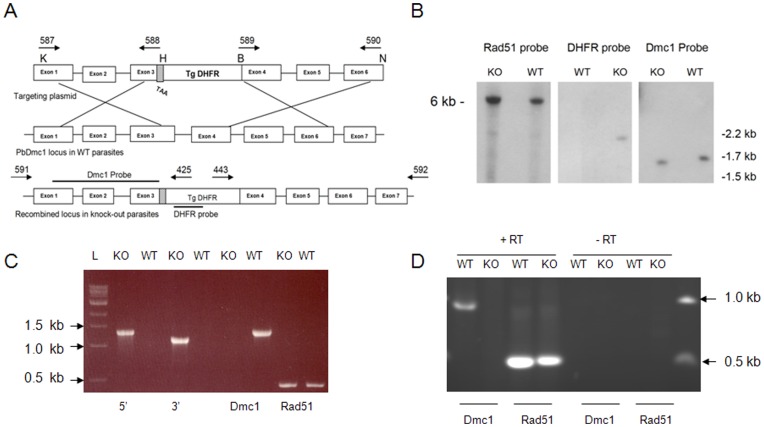
Schematic diagram (not drawn to scale) showing the PbDmc1 targeting disruption plasmid and the resulting locus following double homologous recombination (A). Restriction enzymes used in generating the targeting plasmids are Kpn I (K), Hind III (H), BamHI (B) and Not I (N). The 5′ targeting sequence comprised of exons 1 to 3 and at the end of the exon 3, a stop codon (TAA) was added. The 3′ targeting sequence comprised of exons 4 to 6. The bottom panel shows the resulting locus after recombination events (shown by the X ), where Dmc1 is disrupted with the insertion of the TgDHFR backbone. (B) Southern blot hybridization demonstrated integration at the appropriate locus. The Dmc1 probe hybridized to a 1.5 kb fragment in Dmc1 KO whereas in WT parasites it hybridized to a 1.7 kb fragment following FokI restriction digestion. The DHFR probe hybridized to DNA from Dmc1 KO parasites and not in WT parasites and the Rad51 probe bound to a 6 kb fragment after FokI digestion in both WT and Dmc1 KO parasites suggesting that the Rad51 locus was not affected by disruption of the Dmc1 locus. (C) PCR evidence suggests 5′ and 3′ integration in Dmc1 KO parasites and absence of full length Dmc1. Rad51(control) was present in both WT and Dmc1 KO parasites. L, 1 kb DNA ladder. (D) RT-PCR demonstrated that disruption of Dmc1 abolished expression of full length Dmc1 without any effect on Rad51 expression. Minus reverse transcriptase reactions were negative (data not shown).

Since *P.berghei* is more easily amenable than *P. falciparum* to genetic manipulation, we designed a targeting plasmid to disrupt PbDmc1 and assessed the phenotype of such mutants. Initially we designed a plasmid construct containing 5′ UTR and 3′UTR sequences of PbDmc1 to delete the entire gene, and after at least three failed attempts to generate transformed parasites, we revised our targeting plasmid construct to disrupt the gene The PbDmc1 disruption plasmid contains exons and introns that comprised the 5′ and 3′ targeting sequences (Fig. 1A). Shown in Fig. 1A by the symbol X are the regions where double homologous recombination would occur resulting in the disruption of genomic PbDmc1. Two independent clones obtained from different gene targeting attempts were further characterized genotypically and phenotypically. Diagnostic PCR were done with primer combinations # 591 and # 425 for 5′ integration and primer # 592 and # 443 for 3′ integration and as shown in Fig. 1C, disrupted parasites showed PCR evidence for 5′ and 3′ integration and the absence of full length PbDmc1. Disruption of PbDmc1 was further confirmed by Southern blot analysis which revealed the absence of full length Dmc1 as shown by hybridization of the 5′ targeting sequence probe to an expected shorter fragment (1.5 kb in size) compared to a 1.7 kb fragment in WT parasites (Fig. 1B, Southern data shown for one such clone). The DHFR probe hybridized with DNA from PbDmc1 KO parasites confirming the presence of the targeting sequence containing the DHFR backbone in transformed parasites while there was no signal in WT parasites (Fig. 1B). We further demonstrated that PbDmc1 disruption had no effect on another recombinase, Rad51 which was intact as shown both by PCR (Fig. 1C) and a similar Southern blot profile (6 kb fragment) in WT and PbDmc1 KO parasites (Fig. 1B). The evidence from PCR and Southern blot analysis confirmed disruption of PbDmc1 and data from the Southern blot also ruled out the possibility of episomally maintained plasmid DNA in PbDmc1 KO parasites.

### Loss of Expression of PbDmc1 in Gene Disrupted Parasites

Having demonstrated the disruption of PbDmc1 by both PCR and Southern blot analysis, we further established complete absence of expression of full length PbDmc1 in KO parasites. Transcripts of Dmc1 were examined by RT-PCR and as shown (Fig. 1D), Dmc1 KO parasites did not express Dmc1 whereas WT parasites expressed Dmc1 as shown by a ∼1 kb transcript. We also confirmed that disruption of Dmc1 did not affect expression of Rad51 (see Fig. 1D showing a 0.5 kb Rad51 transcript region expressed in both WT and Dmc1 KO parasites).

### Asexual and Sexual Stages Growth and Transmission Competence of PbDmc1 KO Parasites

We next compared the asexual growth kinetics of PbDmc1 KO parasites to WT parasites. Mice were infected with 10^5^ KO or WT parasites and parasitemia was monitored daily in the infected mice. These studies revealed that the asexual growth kinetics of PbDmc1 KO parasites was comparable to WT parasites (Table 1A). Additionally, when we examined blood smears on the day of mosquito feed (day 4), ratios of male and female gametocytes were comparable between the two groups (Table 1B).

**Table 1:** Asexual growth kinetics and male to female gametocyte ratios.

Day	WT	PbDmc1
3	0.46±0.3	0.59±0.3
4	3.76±0.5	3.09±0.8
5	17.80±5.9	15.02±4.8
6	16.68±7.7	18.77±11.1
7	16.93±5.8	19.90±13.10
Gametocyte ratio (Male: Female)		
**Exp I**	1∶2	1∶2
N	196	216
**Exp II**	1∶2.2	1∶2
N	179	202

Asexual growth kinetics (A) Mice (n = 5) were inoculated with 10^5^ parasites *i.v* and parasitemia (percent infected erythrocytes) is expressed as Mean ± SD. Asexual parasitemias between WT and KO were compared by regression analysis and they were not statistically significant (p = 0.71). (B) Male to female gametocyte ratios of PbDmc1 KO parasites compared to WT parasites when mosquito transmission experiments were done. Gametocytes were counted per 1000 rbc and data shown is from two independent experiments, N is the total number of gametocytes analyzed. Male to female ratios between WT and KO were compared using the Fisher’s exact test and they were not statistically significant (p = 0.99) for both experiments.

**Figure 2:**
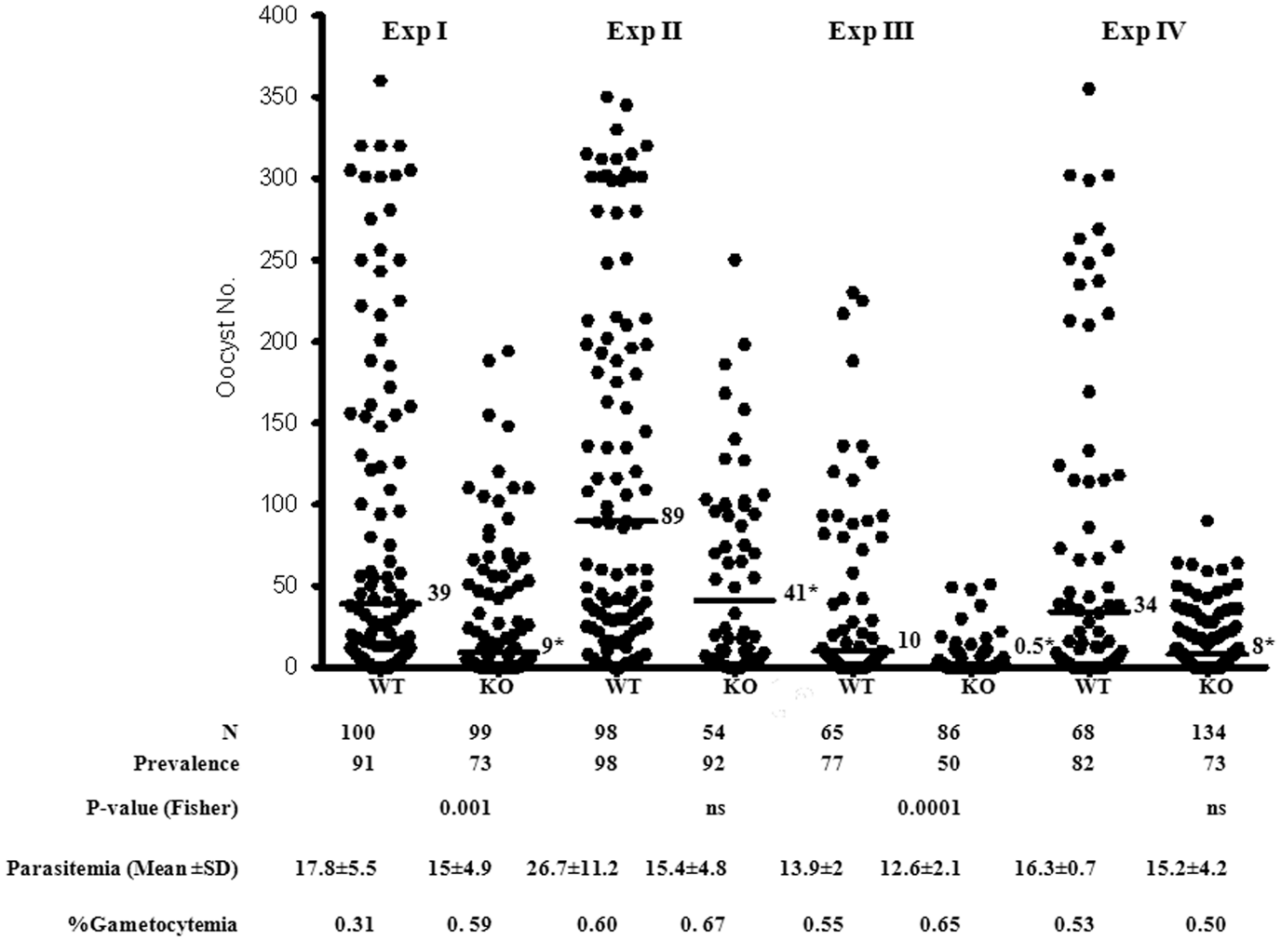
Comparison of WT and PbDmc1 KO oocysts in *An. stephensi* mosquitoes in four independent experiments (Experiments I–III evaluated one of the PbDmc 1 KO clones and Exp. IV represents data for another independent PbDmc 1 KO clone). Horizontal lines represent the median values. Shown below is N, the number of mosquitoes dissected, asexual parasitemia, gametocytemia, the rate of infection (percent of infected mosquitoes). The Fisher’s exact test was used to compare prevalence, i.e the rate of infection between WT and KO and indicated as significant (p<0.05) or not significant (ns). (*Median oocyst numbers of WT were significantly higher than those of PbDmc1 KO parasites, *p*<0.001, Mann-Whitney test).

Since PbDmc1 is a meiotic recombinase and meiotic division occurs early in the mosquito midgut during zygote to ookinete development of the parasites, we then assessed whether transmission to mosquitoes of PbDmc1 KO parasites was in any way affected. The zygote is the only diploid stage of the parasite and soon undergoes meiotic division to produce 4 haploid genomes prior to formation of mature motile ookinetes [2]. To examine transmission competence of PbDmc1 KO parasites, Swiss-Webster mice were infected with parasites (PbDmc1 KO or WT) and 4 days post infection, starved *An. stephensi* mosquitoes fed on the infected mice. Following enumeration of oocysts, PbDmc1 parasites produced 50–80% less oocysts (4 independent experiments with different PbDmc1 KO clones) when compared to WT parasites (Fig. 2). Experiments I-III evaluated one of the PbDmc1 KO clones, while the second PbDmc1 KO clone was tested once (Experiment IV). In addition to the reduction in the mean number of oocysts, PbDmc1 KO parasites showed an average 20% reduction in the percentage of mosquitoes infected in 2 of the 4 experiments performed (Fig. 2). This difference in infection prevalence (percent infected mosquitoes) for the 2 out of 4 experiments was statistically significant (*p*<0.05, Fisher’s exact test). Since the two independent parasite clones revealed similar results on reduced transmission competence as measured by oocyst development (Fig. 2), subsequent studies on further sporogonic development were carried out using one of the two independent PbDmc1 KO clones.

### Sporogonic Development of PbDmc1 KO Parasites

**Figure 3:**
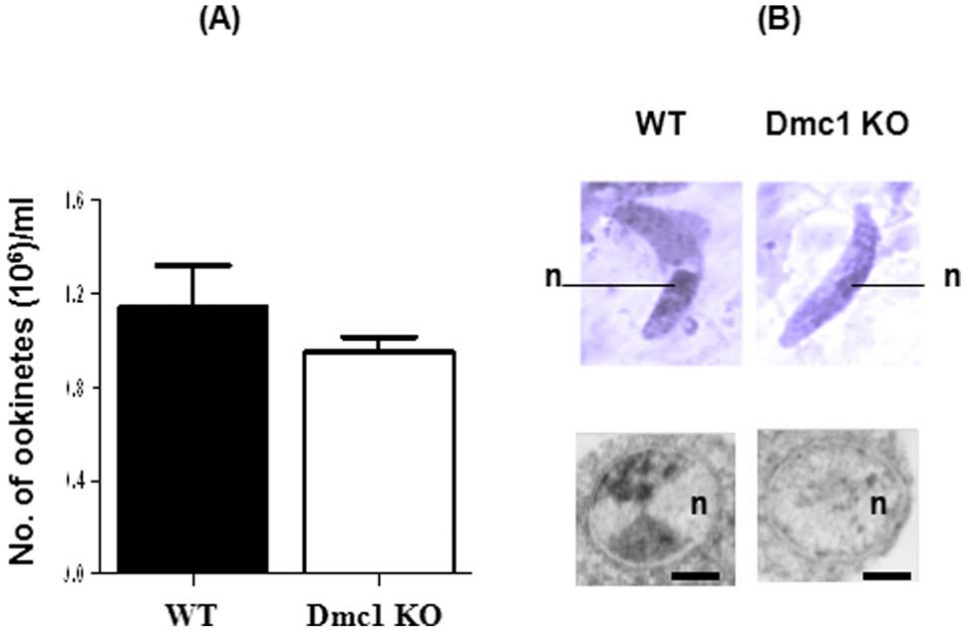
In vitro ookinete counts/ml (mean ± SD) for WT and PbDmc1 KO parasites. Ookinetes cultures were setup from WT and PbDmc1 KO infected mice (n = 3) that had equivalent gametocytemia. Mean ookinete counts were compared using the *t*-test and they were not statistically significant (p>0.05) (A). Morphological comparison by light microscopy of Giemsa stained smears (Upper panel in B) and by TEM of cross-sections of ookinete nuclei showing differences in the chromatin structure (lower panel in B). Fifteen to 22 parasite nuclei were analyzed. n, nucleus. Bars are 100 nm.

Since oocyst development is preceded by ookinete stage development of the parasite, we wondered whether a similar inhibition will be reflected at the level of ookinete formation. Blood from WT or PbDmc1 KO parasite infected mice was cultured and microscopic examination of ookinetes did not reveal any significant difference between WT and Dmc1 KO parasites (Fig. 3A). The morphology (light microscopy) of WT and PbDmc1 KO ookinetes was comparable between the two groups (Fig. 3B). However, TEM examination of ookinetes of WT and Dmc1 KO parasites revealed differences in the nuclei texture. WT parasites showed a normal, condensed chromatin morphology while in the nucleus of PbDmc1 KO parasites, chromatin looked sparse and diffuse, suggesting defects in the packaging of this structure, probably due to defects in meiotic processes (Fig. 3B).

**Figure 4:**
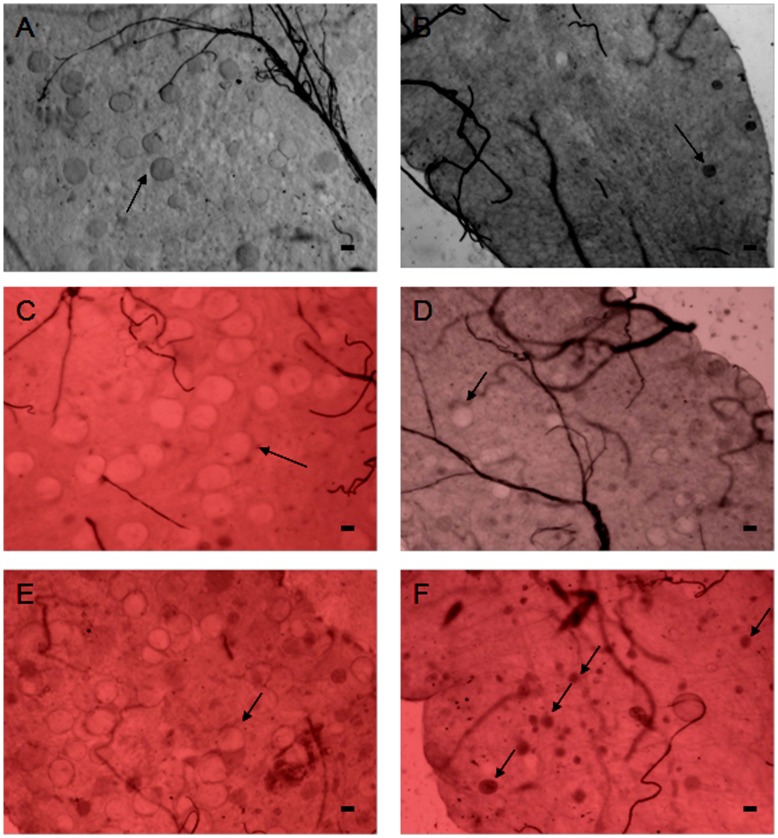
Morphology of WT (A,C,E) and Dmc1 KO (B, D, F) oocyts in the mosquito midgut on day 10 (A, B), 12 (C,D) and 14 (E,F) post blood feeding. Arrows indicate representative oocysts. Scale bar represents 10 µm.

Not only did we find 50–80% reduction in the mean number of oocysts/midgut in PbDmc1 KO parasites but we also observed that PbDmc1 KO oocysts were relatively smaller when compared to WT oocysts (Fig. 4) when examined on different days of mosquito infection. We measured the size of oocysts at day 10 post blood feeding and the average size of WT oocysts (mean diameter±SD, 35.8±8.6 µm) was 1.6 times bigger than the KO oocysts (21.7±4.9 µm). The difference in oocyst sizes between WT and KO was statistically significant (p<0.05 *t*-test).

These observations on defective maturation of oocysts suggested that further sporogonic development might likewise be impaired in PbDmc1 KO parasites.

### Examination of PbDmc1 KO Sporogonic Developmental Stages by Electron Microscopy

**Figure 5:**
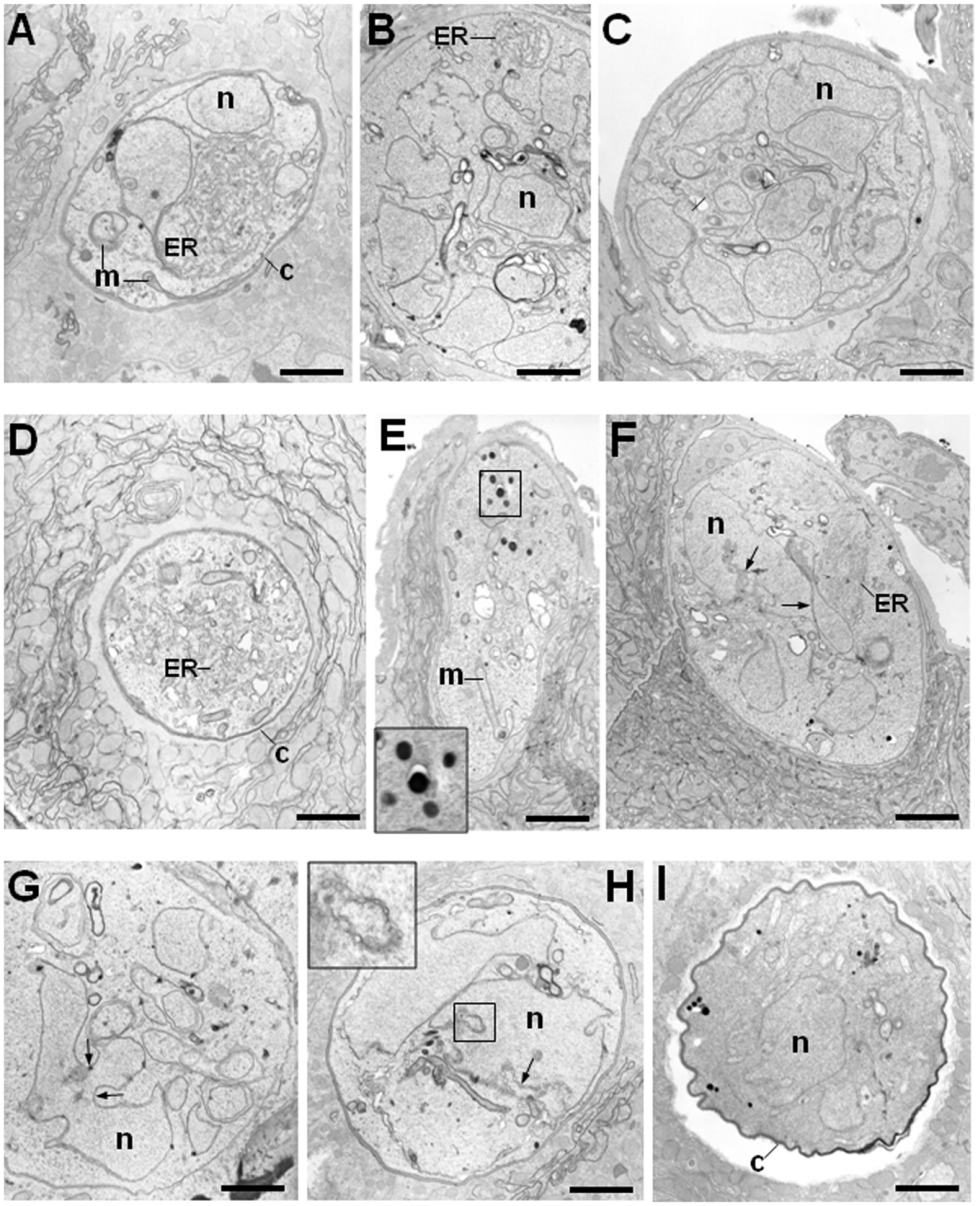
Ultrastructural analysis of WT and Dmc1 KO oocysts 6 days post-infection. TEM of WT (A–C) and Dmc1 KO (D–I) parasites. WT parasites exhibit normal sporogony and contain several nuclei, homogenous in size. By contrast, PbDmc1 KO parasites were smaller in size and show fewer but larger nuclei with aberrant nuclear scission profiles (arrows; inset in H). Accumulation of unknown electron-dense structures as seen in panel E and abnormal capsule morphology in panel I were also characteristics of Dmc1 KO oocysts. c, capsule; ER, endoplasmic reticulum; m, mitochondrion; n, nucleus. Bars are 100 nm.

Oocyst morphology was further inspected by transmission electron microscopy (TEM) to provide ultrastructural details about growth defects in PbDmc1 KO oocysts compared to WT oocysts. Six days post-infection, oocysts of WT parasites were spherical and underwent active schizogony, characterized by multiple fissions of the nuclei (Fig. 5, panels A–C). The endoplasmic reticulum (ER) expanded markedly and reorganized into several dense networks, and the mitochondria became more branched. Confirming data shown in Fig. 4, PbDmc1 KO parasites were smaller in size (panel D) or still elongated (panel E). Sporogony in KO parasites was slower as a limited number of nuclei were observed in any sections. Large bilobed nuclei (panels F–H, arrows) and endomitosis structures within the nucleus (inset in panel H) were identified, indicative of defects in karyokinesis in PbDmc1 KO parasites. Interestingly, these KO parasites also exhibited two other distinct phenotypes: the accumulation of electron-dense granules of unknown origin (panel E), and a retracted and convoluted capsule surrounding the parasite (panel I).

**Figure 6:**
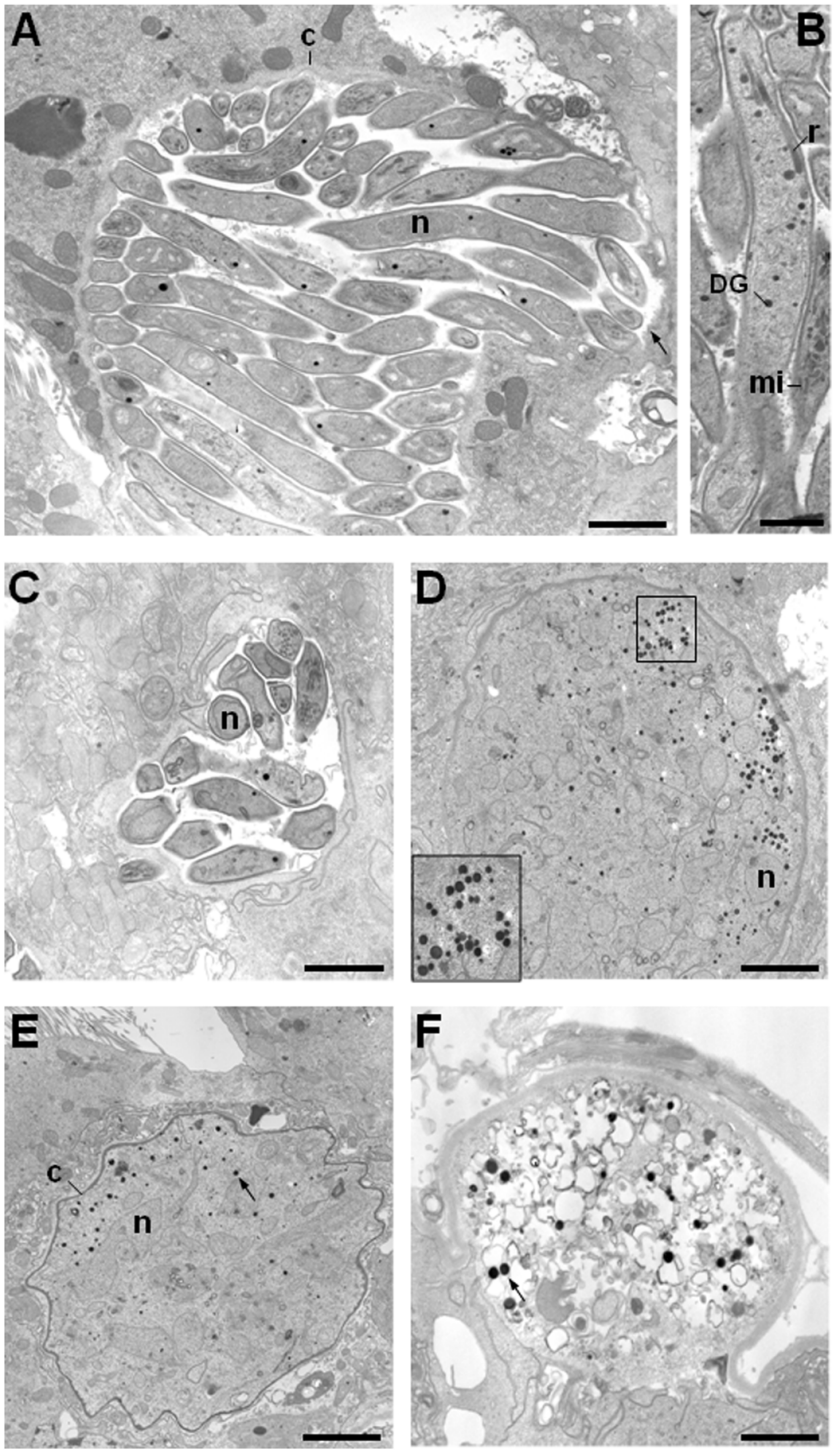
Ultrastructural analysis of WT and PbDmc1 KO oocysts 13 days post-infection TEM of WT (A,B) and mutant (C–F) parasites. From WT oocysts, mature sporozoites were visible and ready to escape midgut epithelial cells after rupture of the capsule (arrow in A) before migration to the salivary glands. Few PbDmc1 KO were also able to form sporozoites (panel C), but a majority of the oocysts were still undergoing sporogony (panel D–F) and some oocysts looked highly vacuolated (panel F). Note the increasing number of electron-dense structures in panels D to F and the abnormal shape of the capsules for some oocysts (panel E) as observed 6 days post-infection.c, capsule; DG, dense granule; mi, microneme; n, nucleus. Bars are 2 µm (A,C), 1 µm in B and 250 nm in D–F.

The sporozoite stage is generated by budding from the multinucleate oocyst in the mosquito midgut starting from day 12 after infection. Figure 6 illustrates the formation of mature sporozoites from large WT oocysts (panels A–B) 13 days post-infection. These stages were competent to invade the salivary glands since only a few parasites were detectable in the midgut (panels A–B) beyond day 13. In contrast, PbDmc1 KO parasites were still visible in midgut tissues in accordance to an abnormal growth phenotype. Three different types of oocysts were distinguishable: 1) like WT parasites, oocysts containing fully-formed sporozoites (∼10%; panel C); 2) late oocysts which had not yet subdivided into sporoblasts and strongly encapsulated (∼20%; panels C and D), and 3) apparently dying oocysts, largely vacuolated, containing unidentifiable structures (∼70%; panel F). Additionally, as the oocyst replication and maturation occurred, the number and size of the electron-dense granules also appeared to increase. Twenty-seven days after-infection, WT parasites were abundantly found in salivary ducts and glands (Fig. S2). However, it was not possible to analyze the organellar composition of Dmc1 KO sporozoites in salivary glands due to undetectable numbers of sporozoites in the salivary glands.

### Kinetics of Sporozoite Development in the Mosquito

Further experiments were designed to assess the kinetics of sporozoite development in midguts and salivary glands. In two experiments, PbDmc1 KO parasites produced fewer midgut sporozoites between day 11 and day 14 with a peak around day 19–21 (Table 2). In contrast mosquitoes infected with WT parasites contained 30-fold higher midgut sporozoites when compared to PbDmc1 KO parasites. When salivary gland were examined at day 21, WT sporozoites were detected at approximately 6,000 per mosquito whereas PbDmc1 KO sporozoites were approximately 20 times fewer in number when compared to WT. Though the number of sporozoites increased by day 28, they were still fewer when compared to WT (Table 2). On day 28, the difference of sporozoite counts between WT and PbDmc1 KO parasites was modest in one experiment but more pronounced in a second one. However, the proportion of PbDmc1 KO parasites in salivary glands versus the parasite population in the midgut was always lower as compared to WT. Another interesting observation was that oocysts of PbDmc1 KO persisted in at least 70% of the midguts up to day 35 post blood feeding whereas in WT parasites almost all the midgut oocysts had disappeared. Our results suggest that there is aberrant development in the maturation of PbPbDmc1 KO sporozoites as shown by delayed kinetics of midgut and salivary gland sporozoites when compared to WT. Although the number of sporozoites in Dmc1 KO mosquito salivary glands was markedly lower, we assumed that they would be biologically functional, i.e transmission competent. In order to test for functional competence of those sporozoites, approximately 100 PbDmc1 KO or WT infected mosquitoes at 21 or 28 days post blood feeding were allowed to feed on naive mice and blood stage infection monitored by microscopic examination of blood smears. In three independent experiments PbDmc1 KO sporozoites failed to establish patent parasitemia whereas WT sporozoites were infectious to naive Swiss-Webster mice and mice became parasitemic on days 5 to 7 (Table 3). In parallel, we injected 4,000 salivary gland sporozoites i.v in mice and no patent parasitemia was observed in PbDmc1 KO inoculated mice whereas in WT infected mice, blood stage parasites were observed within 5–7 days. Additional experiments were conducted to assess the ability of PbDmc1 KO sporozoites to invade hepatocytes. WT or Dmc1 KO salivary gland sporozoites were incubated with a hepatocyte cell line (Hepa 1–6) and inside/outside staining was performed using an anti-CS antibody to enumerate sporozoites that were either internalized or remained extracellular. These studies suggested that Dmc1 KO sporozoites are markedly deficient in their ability to invade hepatic cells since approximately 1.3% of the Dmc1 KO sporozoites were found inside hepatocytes (2 inside/151 outside) as compared to 60% inside for WT sporozoites (63 inside/105 outside). The data suggests that Dmc1 KO parasites can still infect a hepatocyte cell line, but much less efficiently than the wild type.

**Table 2:** Kinetics of sporozoite development in midguts and salivary glands of mosquitoes.

		No. of sporozoites per mosquito			
		WT		PbDmc1	
Exp	Day	Midgut	Salivary gland	Midgut	Salivary gland
**I**	11	4,000	nd	148	nd
	14	12,800	nd	387	nd
	19	38,580	nd	2,266	nd
	21	12,827	5,655	1,750	375
	28	3,000	1,000	1,515	675
**II**	14	9,000	nd	0	nd
	21	1,000	6,109	2,853	133
	28	13,800	6,466	933	1,612
	35	3,600	7,000	1,875	1,219

On each day indicated above, pools of 15–30 mosquitoes were dissected and sporozoite counts made on a hemocytometer. nd, not determined.

**Table 3:** Infectivity of WT and PbDmc1 KO sporozoites in mice.

Infection	Pre-patent period	Number infected
WT^a^	5–7 days	0/9
PbDmc1^a^	−	0/9
WT^b^	5–7 days	0/6
PbDmc1^b^	−	0/6

Data for mouse mosquito infections is pooled for three independent experiments and data for mouse i.v inoculations is pooled for two independent experiments.

a Mice were exposed to 100 infected mosquitoes for 45 minutes.

b Mice were injected i.v (tail vein) with 4,000 salivary gland sporozoites.

### Susceptibility of PbDmc1 KO Parasites to Bizelesin, a DNA Damaging Drug

Bizelesin is a DNA damaging drug that produces DNA interstrand cross-linking following alkylation of the N3 position of adenine on opposite DNA strands [22], [23]. Since Dmc1 plays a role in meiotic recombination and maintaining DNA stability [9], we evaluated susceptibility of PbDmc1 KO parasites to different concentrations of bizelesin. Initial experiments to optimize and assess susceptibility of PbDmc1 KO parasites indicated that concentrations of 10 µg/kg or 15 µg/kg bizelesin administered to mice 12 or 24 hr prior to mosquito transmission resulted in nearly 100% inhibition of oocyst formation (data not shown). We then tested doses of 2.5 and 5 µg/kg bizelesin which resulted in approximately 85% oocyst inhibition in WT parasites. In order to further compare susceptibility differences between WT and Dmc1 KO parasites, bizelesin was evaluated at concentrations of 1 µg/kg and below. PbDmc1 KO parasites in mice succumbed more to the effects of bizelesin as measured by inhibition of subsequent oocyst formation compared to WT parasites (Table 4). For example, at 0.5 µg/kg bizelesin, Dmc1 KO parasites were inhibited by a magnitude of approximately 70% as compared to only 10% inhibition observed with WT parasites. We calculated the median number of oocysts in the mosquitoes that fed on mice treated with different concentration of bizelesin and infected either with WT or Dmc1 KO parasites. These data were then used to calculate bizelesin concentration that would result in 50% inhibition (IC_50_) of oocyst formation and the IC_50_ were 5.2 µg/kg and 0.28 µg/kg for WT and PbDmc1 KO parasites, respectively. Thus these data suggest that PbDmc1KO parasites were approximately 18 times more susceptible to bizelesin when compared to WT, implying that Dmc1 may play a crucial role in maintaining DNA stability and integrity during sexual development of *P. berghei* parasites. Taken together our results argue for an essential role for PbDmc1 during sexual development, i.e formation of oocysts and subsequent sporogonic development into transmission competent infectious sporozoites.

**Table 4:** Susceptibility of PbDmc1 KO parasites to the DNA alkylating agent bizelesin.

	WT					PbDmc1 KO				
Drug Conc. (µg/kg)	0	0.1	0.5	1	10	0	0.1	0.5	1	10
Median oocysts	41	68	40	41	0	23	20	3	1	0
Range	0	0	0	0	0	0	0	0	0	0
	370	358	350	231	8	148	88	66	30	0
25 percentile	4	6	9	14	0	6	6	0	0	0
75 percentile	122	198	99	76	0	42	33	11	8	0
N	76	99	97	107	124	86	86	79	91	120
% Inhibition	–	0	2.4	0	100	–	13	87	96	100

Swiss Webster mice were infected with WT or PbDmc1 KO parasites and 12 hours prior to transmission, mice were treated with varying concentrations of bizelesin and starved mosquitoes fed on the mice. Shown are the median, range (min and max values), 25 and 75 percentiles and % inhibition which was calculated by dividing median number of oocysts at 0.1, 0.5, 1 and 10 µg/kg Bizelesin by the number of oocysts from mice that received vehicle only. N represents total number of mosquitoes analyzed. (* Differences in the oocyst numbers between WT and PbDmc1 KO parasites were statistically significantly, *p*<0.05, Kruskal-Wallis test).

## Discussion

Dmc1 is a bacterial RecA homolog widely conserved among different species, for example PbDmc1 and PfDmc1 and HsDmc1 share 53% similarity at the amino acid level. In *Plasmodium*, meiosis occurs in the diploid zygote stage and the subsequent stages are haploid throughout the life cycle. In view of the central role of meiosis in sexual development of *Plasmodium*, we examined the role of PbDmc1, a meiotic specific recombinase by targeted gene disruption approach. Kinetics of asexual and sexual development, including male to female ratios were comparable between WT and Dmc1 KO parasites. However, PbDmc1 KO parasites formed fewer oocysts that were smaller in size compared to WT. While some heterogeneity has been observed in the size of oocyst in mosquito midguts [2], our observations clearly showed that PbDmc1 KO oocysts were relatively smaller when compared to WT. Ultrastructural evidence revealed that some of the smaller oocysts were devoid of nuclei and other organelles such as mitochondria and endoplasmic reticulum. While the actual mechanisms are not known, this could have been caused by aberrant meiotic recombination leading to improperly differentiated oocysts. Additionally, we do not know if PbDmc1 KO oocysts are more susceptible to the mosquito immune system [2] resulting in degenerate oocysts that we observed. It is also intriguing to note that PbDmc1 KO oocysts persisted up to at least day 35 in the mosquito midgut when WT oocysts had cleared. Dmc1 may thus be involved in normal development of parasite sexual stages inside the mosquito.

The number of sporozoites produced in PbDmc1 KO infected mosquitoes were fewer in number compared to WT and these sporozoites were not infections to naive mice. The lack of infection could be due to defects in sporozoites formation as a result of Dmc1 disruption or that the sporozoite density failed to reach a critical threshold at any given point required for a productive infection in mice. To support this argument, in PbTRAP knockouts, 100,000 sporozoites were required to produce a blood stage infection in rodents [24]. Additionally, the less efficiency of PbDmc1 KO sporozoites as compared to WT sporozoites to invade hepatocyte *in vitro* may also explain the failure of PbDmc1 KO sporozoites to productively infect mice in direct mosquito feeding experiments. Disruption of PbDmc1 adversely affected mosquito stage development of parasites as shown by small degenerate oocysts and lower production of sporozoites that failed to infect naive mice. These findings suggests a critical role for Dmc1 in sporogonic development and concur with studies in mice which showed that Dmc1 mutants were sterile [25].

It is worth mentioning that there is a possibility that the effect of disrupting Dmc1 seen here is a result of experimental manipulation. However, considering that two independent clones obtained from two independent transfection experiments tested in these studies had a similar phenotype, and that it takes less than 3–4 weeks to generate the knock out clones, it is unlikely that defective sporogonic development could have been due to random insertion of the targeting construct or a result of prolonged asexual growth period for selecting and cloning KO parasites.

Dmc1 may also play an important role in DNA stability and repair as suggested by our experiments with a DNA damaging drug, bizelesin which revealed that Dmc1 deficient parasites were 18 times more susceptible to bizelesin when compared to WT parasites. In yeast, Dmc1 mutants have deficiencies in regulating processing of the 5′ strand of DSB which may affect stability of DNA [9], [26], [27]. The role for Dmc1 in maintaining DNA stability is not surprising given that Rad51 is upregulated in *P. falciparum* and *T. brucei* in response to DNA insults by chemical agents such as MMS [28], [29]. It is well known that Dmc1 and Rad51 operate cooperatively during meiosis however, we do not know whether Rad51 could partially compensate Dmc1 deficiency in our KO parasites. In studies reported here, disruption of Dmc1 did not completely abrogate development and differentiation of zygotes to ookinetes and oocysts. Ookinete numbers were comparable between KO and WT. At the oocyst stage, though most of the oocysts were smaller in size there were some oocysts that looked like normal WT oocysts suggesting some repair mechanism compensating for the absence of Dmc1. There is a remote possibility that Dmc1 disrupted parasites expressed a truncated sequence at the N-terminal domain that could have retained the ability to bind DNA without the ATPase (catalytic) domain and this could have provided some partial DNA stabilizing functions. Another possibility is that there could be a redundant pathway dependent or independent of Rad51 that could be activated to repair DNA in KO parasites. In future it would be interesting to examine whether Rad51 or other DNA repair molecules are over expressed in Dmc1 KO parasites to compensate for Dmc1 deficiency.

Bizelesin inhibited transmission of both WT and KO parasites at 10 µg/kg and this suggests that bizelesin has potent transmission reducing effects on *Plasmodium*. Indeed a previous study has shown that drugs that damage DNA like centanamycin can block transmission of parasites to mosquitoes [30]. Adozelesin, a drug related to bizelesin inhibited growth of *P. falciparum in vitro* at pico molar concentrations and completely inhibited a blood stage infection of *P. chabaudi* in mice at 25 µg/kg [30]. Though this class of DNA binding and DNA alkylating drugs may offer alternate ways to treat malaria or reduce transmission, bizelesin and other related compounds have been shown to exert cytotoxicity when evaluated as cancer therapeutic agents [31], [32]. In any case, these drugs offer valuable research tools to investigate DNA damage response pathways in *Plasmodium*.

Among protozoan parasites, Dmc1 has been characterized in *Trypanosoma brucei* by gene knockout studies [29]. Unlike *Plasmodium* Dmc1, *T. brucei* Dmc1 did not play a critical role in DNA repair, recombination or antigenic variation [29]. This discrepancy may be partially explained by the fact that bloodstream *T. brucei* parasites employ mitotic and not meiotic division during infection in the mammalian host [29].

In conclusion, our current studies revealed that Dmc1 plays an essential role in sporogonic development of *Plasmodium* in the mosquito as shown by a reduction in oocyst numbers and prevalence of infected mosquitoes. Moreover, there was a defect in the development of midgut sporozoites to salivary gland sporozoites and this could affect transmission of Dmc1 KO parasites given that the average life span of mosquitoes is about 3 weeks.

## Materials and Methods

### Ethics Statement

All experiments with mice were done as per the protocol (MO07H4) approved by the Institutional Animal Use and Care Committee of the Johns Hopkins University.

### *P. berghei* Transfections

To generate *P. berghei* parasites lacking PbDmc1, the pB3D plasmid (Courtesy of Andy Waters) was digested with KpnI and HindIII and a 567 bp fragment consisting of the 5′ region of PbDmc1, nt position +26 to +593 was ligated between the KpnI and HindIII restriction sites. The primers used to amplify this fragment were #587, 5′-ggtaccCAGCCAGCAAAGTTGCAT-3′ and #588, 5′-aagcttCTACACTTGAGTTTTTCCGCA-3′. The resulting plasmid containing the 5′ targeting sequence was then digested with BamHI and NotI and a 3′ targeting sequence 490 bp in size, nt position +980 to nt +1470 (comprising of exons 4 through 6 and the spanning introns) was cloned into the BamHI and NotI restriction sites. The primers that were used to amplify this fragment were # 589, 5′- gaattcCGAGTAGACTTTAGTGGGC -3′ and # 590, 5′-gcggccgcCGGGAAGGTTTGGAG -3′. The plasmid construct (Fig. 1A) was verified both by PCR and sequencing. For transfection studies, plasmid DNA was purified using the plasmid Maxi kit (Qiagen) and digested with KpnI and NotI to obtain the targeting cassette. Ten µg of targeting DNA was used for each transfection experiment using the Amaxa Human T cell nuclear factor kit with the U033 settings. Transfected parasites were injected *i.v* into naive Swiss-Webster mice and 24 hr post transfection, mice were put on drinking water containing pyrimethamine (0.07 mg/ml). Drug resistant parasites were genotyped by PCR and cloned by limiting dilution as previously described [33]. Parasite clones from two independent tarnsfections were selected and investigated further.

### Diagnostic PCR and Southern Blot Analysis of PbDmc1 KO Parasites

Parasites resistant to pyrimethamine were genotyped to characterize double homologous recombination at the PbDmc1 locus. Diagnostic PCR for 5′ integration was performed with a forward primer, #591, 5′- GCTTTCTTAATTTGGCGTTG-3′ located 456 bp upstream of the beginning of the 5′ targeting sequence and a reverse primer (#425, 5′-GAGTTCATTTTACACAATCC-3′) located on the DHFR backbone. Likewise, the diagnostic PCR for 3′ integration was performed using a forward primer (#443, 5′-CAATGATTCATAAATAGTTGG-3′) located on the DHFR backbone and a reverse primer (#592, 5′-ATTTTGTTCATTCTTTATATGTC-3′) positioned 549 bp downstream from the 3′end of the targeting sequence. Two parasite clones were obtained from two independent transfection experiments.

Further molecular characterization of transformed parasites was done by Southern blot analysis. Briefly, 5 µg DNA from WT and PbDmc1 KO parasites was digested with FokI, ran on a 1% agarose gel, DNA transferred onto a nitrocellulose membrane and probed with the PbDmc1 5′ targeting sequence, a DHFR probe and full length PbRad51 used as control (Fig. 1A). All probes were generated by PCR, and labeled with 50 µCi (^32^P dCTP from Perkin Elmer) and hybridizations were performed as previously described [33].

### Reverse Transcriptase PCR to Examine Expression of PbDmc1

To investigate whether full length PbDmc1 was no longer expressed in the PbDmc1 KO parasites, total RNA was extracted from ∼200 µl blood drawn from WT or PbDmc1 KO infected Swiss-Webster mice using the RNeasy Qiagen kit. RNA was reverse transcribed using the Qiagen Omniscript kit to obtain cDNA with the antisense primer (primer #590) for PbDmc1 and the antisense primer (primer #611, 5′- AAGCTTTTACATTTACCCTCACC -3′) for PbRad51 (control) in a 20 µl reaction containing 3 µl of RNA template, 500 µM each deoxynucleotide, 10 units RNase inhibitor, 1X reverse transcription (RT, Qiagen) buffer, 0.5 µM of antisense primer and 4 units of Omniscript reverse transcriptase (Qiagen). The reaction was incubated at 37°C for 1 hr and 2 microliters from this RT step was used as template for the PCR in a 25 µl reaction mix containing buffer (50 mM KCl, 10 mM Tris-HCl pH 8.3, 1.5 mM MgCl_2_), 100 µM of each deoxynucleotide, 0.4 µM of each primer (# 587 and # 590 for PbDmc1; #610, 5′-GGTACCGCTAA CGCAAAAGAAG-3′ and # 611 for Rad51) and 1.25 U *Taq* polymerase. The PCR cycling conditions were as follows; initial denaturation at 94°C for 2 min, 45 cycles of denaturation at 94°C for 30 seconds, annealing at 50°C for 35 seconds, and extension at 68°C for 2 ½ min. All the samples were tested without the reverse transcriptase to rule out the possibility of amplification from DNA instead of RNA template.

### Characterization of Asexual Growth Kinetics, Ookinete Formation and Transmission to Mosquitoes of PbDmc1 KO Parasites

PbDmc1 KO parasites were compared to WT parasites for their asexual growth kinetics and transmissibility to *An. stephensi* mosquitoes. Swiss-Webster mice (female, 5–8 weeks old) were infected with approximately 10^5^ WT or PbDmc1 KO parasites *i.v* (5 mice/group). Parasitemia was monitored daily by examining Giemsa stained thin blood smears.

In order to assess transmission to mosquitoes, Swiss-Webster mice were infected with 10^6^ parasites (WT or PbDmc1 KO) and on day 4 post infection, starved *An. stephensi* female mosquitoes were allowed to feed on the infected mice, anesthetized with Avertin. Initially, both the Dmc1 KO clones were tested in such mosquito infection experiments. Blood fed mosquitoes were maintained at 19°C, 80% relative humidity and 10 days post-feeding, mosquito midguts were dissected to enumerate oocysts. Additional experiments were performed to compare ookinete stage development in PbDmc1 KO and WT parasites. Briefly, Swiss-Webster mice infected with PbDmc1 KO or WT parasites with equivalent gametocytemia were bled and the blood diluted 1∶5 in RPMI supplemented with 10% fetal bovine serum and incubated at 19°C for 24 hr as previously described [33]. Thin smears were prepared and stained with Giemsa to examine the morphology of ookinetes. To enumerate ookinetes, 15 µl of the culture was placed on a hemocytometer and examined under the light microscope at 400x magnification and counts expressed as ookinetes per ml. For electron microscopy analysis, ookinetes cultured as described above were spun at 6,500 rpm for 5 min and RPMI media discarded. RBC were lysed by adding 1 ml of lysis buffer (0.15 M NH_4_Cl, 10 mM KHCO_3_, 0.1 mM EDTA) for 5 min on ice. Ookinetes were washed 3 times with PBS and resuspended in 100 µl PBS and samples for electron microscopy were prepared as described below.

### Morphological Evaluation of PbDmc1 KO Oocysts and Kinetics of Sporozoite Development

PbDmc1 KO and WT oocysts on day 10, 12 or 14 were stained with 0.1% mercurochrome and examined under the light microscope at 100x magnification to compare the size of oocysts between WT and PbDmc1 KO parasites. The size of oocysts at day 10 post blood feeding were measured with the aid of the Image Pro Plus software (Media Cybernetics, MD). Kinetics of sporozoite development in WT and Dmc1 KO parasites was assessed in midguts and salivary glands dissected at various time points. For sporozoite kinetics, midguts were dissected from 15–30 mosquitoes and placed in approximately 300 µl PBS, and homogenized in an eppendorf tube using a plastic pestle. The tube was spun at 1,000 rpm for 1 min and sporozoites in the supernatant were counted using a hemocytometer and expressed as number of sporozoites per midgut. For salivary gland sporozoites, 15–30 salivary glands were isolated and processed similar to midgut tissue samples.

#### Infectivity of Dmc1 KO sporozoites to mice and a hepatocyte cell line (Hepa 1–6)

In order to examine the ability of PbDmc1 KO sporozoites to infect mice, 100 mosquitoes with salivary gland sporozoites fed on anesthetized mice for 45 min and thin blood smears were prepared daily to monitor blood stage parasitemia. Additional experiments were performed where 4,000 WT or PbDmc1 KO salivary gland sporozoites were injected (i.v.) into each mouse and parasitemia monitored as described above.

In order to examine the ability of PbDmc1 KO sporozoites to infect hepatocytes, we performed *in vitro* invasion assays using a hepatocyte cell line (Hepa 1–6). Hepa 1–6 cells were cultured for approximately 72 hours in Lab-tek 8 well chamber slides (Nunc, Naperville, IL). Approximately 6,000 sporozoites (WT or Dmc1 KO) were added to each well and slides spun at 2,860 rpm for 5 min. Slides were then incubated at 37°C for 2 hrs. Slides were washed 3 times with PBS for 5 min to remove unbound sporozoites and fixed with 4% paraformaldehyde/0.02% glutaraldehyde for 15 min at RT. Slides were washed twice with PBS and blocked with 3%BSA/PBS. Anti-CS antibody (3D11) was added at a dilution of 1∶1,500 and cells incubated for 1 hr at 37°C followed by 3 washes as described above. Anti-mouse IgG TRITC diluted 1∶1,000 was added to the wells and incubated at RT for 1 hr. To permeabilize cells, slides were washed 3x with PBS and fixed with 100% methanol for 15 min. After permeabilization, methanol was removed and wells were washed and blocked at RT for 1 hr. Anti-CS was added to the wells and incubated at 37°C for 1 hr. After the washing step, anti-mouse IgG FITC was added at 1∶1,000 dilution and incubated at RT for 1 hr. After the final wash, slides were stained with DAPI for 5 min, mounted with coverslips and examined under the Nikon E800 fluorescent microscope to score for intracellular (FITC) and extracellular (TRITC/FITC) sporozoites.

### Electron Microscopy

For thin-section transmission electron microscopy (TEM), ookinetes or mosquito tissues containing WT and PbDmc1 KO parasites were fixed in 2.5% glutaraldehyde (Electron Microscopy Sciences; EMS) in 0.1 M sodium cacodylate buffer (pH 7.4) for 1 h at room temperature. They were washed 3 times in 0.1 M cacodylate buffer and then postfixed for 1 h in 1% osmium tetroxide (EMS) in the same buffer at room temperature. After 3 washes in water the samples were stained for 1 h at room temperature in 2% uranyl acetate (EMS), then washed again in water and dehydrated in a graded series of ethanol. The samples were then embedded in Embed-812 epoxy resin (EMS). Ultrathin (50–60 nm) sections were cut using a Reichert Ultracut ultramicrotome and collected on formvar- and carbon-coated nickel grids, stained with 2% uranyl acetate and lead citrate before examination with a Philips 410 Electron Microscope (Eindhoven, the Netherlands) under 80 kV.

### Susceptibility of PbDmc1 Parasites to the DNA Alkylating Drug Bizelesin

We evaluated susceptibility of PbDmc1 KO parasites to different concentrations of bizelesin (a gift from the Drug Synthesis and Chemistry Branch, Developmental Therapeutics Program, Division of Cancer Treatment and Diagnosis, National Cancer Institute, USA). Mice (n = 3) were infected with 10^6^ WT or PbDmc1 KO parasites and 12 or 24 hr before transmission to mosquitoes, mice were injected *i.p* with 0.2 ml containing bizelesin concentrations ranging from 0.1 µg to 15 µg/kg. Bizelesin was prepared in a vehicle of 10% cremaphor, 2% N, N-dimethylacetamide, and 88% water at 2 mg/ml. Control mice were given 0.2 ml of the vehicle alone. Following drug treatment, starved *An. stephensi* mosquitoes were allowed to feed on the treated mice and oocysts were enumerated as described above.

### Statistical Analyses

Statistical analyses were performed with the aid of Graphpad Prism Software (Graphpad Software Inc, CA). For asexual kinetics, regression analysis was used to examine differences between WT and KO parasites. Oocysts median counts between WT and KO were compared using the Mann-Whitney U test. The Fisher’s exact test was used to examine differences in prevalence (rate of infection) in WT and PbDmc1 KO infected mosquitoes. P values less than 0.05 were considered statistically significant. To compare the effect of bizelesin on oocyst development, the distributions of oocyst numbers between WT and KO were analyzed using the Kruskal-Wallis test and values less than 0.05 were considered statistically significant.

## Supporting Information

Figure S1 **A schematic diagram showing sexual and sporogonic development of** ***Plasmodium*** **parasites in the mosquito vector.** M (male); F(female); vertical double headed arrow separates malaria parasite sexual stages in the vertebrate host (to the left of the arrow) and sexual and sporogonic stages in the anopheline mosquito vector (to the right of the arrow): * denotes the stage of meiotic division.
(TIF)

Figure S2 **Detection of** ***P. berghei*** **WT in mosquito salivary glands 27 days post-infection.** A–B. Electron micrographs. After egress from mosquito gut tissues, mature sporozoites (arrow) were observed in salivary ducts (SD). Parasite organelles involved in motility (IMC, inner membrane complex), invasion (mi, micronemes) and parasitophorous vacuole (PV) establishment and transformation (r, rhoptries; DG, dense granules) necessary for mammalian liver infection, are identified. Compared to WT parasites, eight-times more grids were scrutinized for DmcI KO samples but no parasites were found in any sections.
(TIFF)

Table S1 **Homolog of PbDmc1 with Dmc1 protein (amino acid) in other species.**
(DOC)
