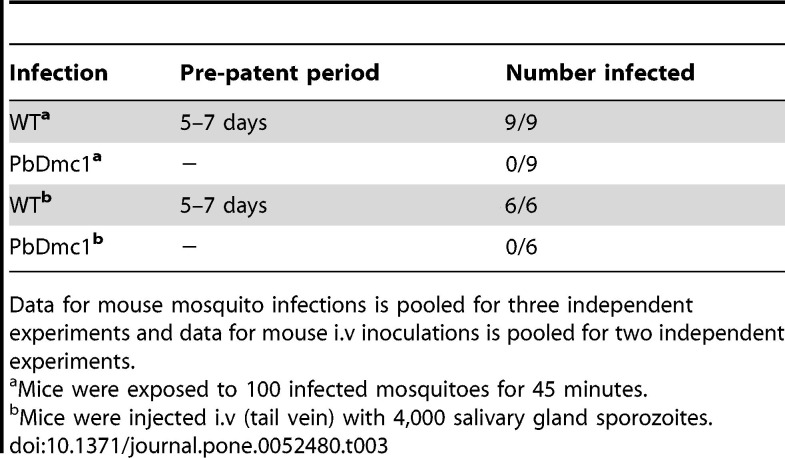# Correction: Aberrant Sporogonic Development of Dmc1 (a Meiotic Recombinase) Deficient *Plasmodium berghei* Parasites

**DOI:** 10.1371/annotation/7d47c32e-7ede-4158-a801-db445abbd2b7

**Published:** 2013-10-01

**Authors:** Godfree Mlambo, Isabelle Coppens, Nirbhay Kumar

In Table 3, the "Number infected" column contains two errors. WT^a^ should be 9/9 and WT^b^ should be 6/6. Please see the corrected Table 3 here: 

**Figure:**