# Supplementary material for: Aberrant Sporogonic Development of Dmc1 (a Meiotic Recombinase) Deficient Plasmodium berghei Parasites
Source: PLoS One. 2012 Dec 21;7(12):e52480. doi: 10.1371/journal.pone.0052480 (PMC3528682; doi:10.1371/journal.pone.0052480)
Supplement: Table S1 — Homolog of PbDmc1 with Dmc1 protein (amino acid) in other species. (DOC) [file pone.0052480.s003.doc]

Table S1. Homolog of PbDmc1 with Dmc1 protein (amino acid) from other species.

|  | PbDmc1 | PcDmc1 | PfDmc1 | PkDmc1 | PvDmc1 | PyDmc1 | ScDmc1 | HsDmc1 |
| --- | --- | --- | --- | --- | --- | --- | --- | --- |
| PbDmc1 | 100 | 54 | 88 | 96 | 100 | 80 | 48 | 53 |
| PcDmc1 |  | 100 | 52 | 54 | 54 | 56 | 27 | 30 |
| PfDmc1 |  |  | 100 | 86 | 88 | 77 | 45 | 53 |
| PkDmc1 |  |  |  | 100 | 96 | 79 | 48 | 53 |
| PvDmc1 |  |  |  |  | 100 | 80 | 48 | 53 |
| PyDmc1 |  |  |  |  |  | 100 | 42 | 46 |
| ScDmc1 |  |  |  |  |  |  | 100 | 51 |
| HsDmc1 |  |  |  |  |  |  |  | 100 |

The accession numbers are PbDmc1, XP_679989; PcDmc1, XM_731684; PfDmc1, AAK43698; PkDmc1, XP_002258170; PvDmc1, XP_00161; PyDmc1, XP_726066; ScDmc1, AAA34571; HsDmc1, NP_008999. Pb (*Plasmodium berghei*), Pc (*P. chabaudi chabaudiI*); Pv (*P. vivax*), Hs (*Homo sapiens*), Sc (*Saccharomyces cerevisiae*), Py (*P. yoelii*), Pf (*P. falciparum*) and Pk (*P. knowlesi*),
